# Outcomes after Microvascular Decompression for Hemifacial Spasm without Definite Radiological Neurovascular Compression at the Root Exit Zone

**DOI:** 10.3390/life13102064

**Published:** 2023-10-16

**Authors:** Chiman Jeon, Minsoo Kim, Hyun-Seok Lee, Doo-Sik Kong, Kwan Park

**Affiliations:** 1 Department of Neurosurgery, Korea University Ansan Hospital, Ansan 15355, Republic of Korea; caudate@daum.net; 2 Department of Neurosurgery, Gangneung Asan Hospital, University of Ulsan College of Medicine, Gangneung 25440, Republic of Korea; kimminsoo@fastmail.com; 3 Department of Neurosurgery, Konkuk University Medical Center, Seoul 05030, Republic of Korea; kwanpark@skku.edu; 4 Department of Neurosurgery, Samsung Medical Center, Sungkyunkwan University School of Medicine, Seoul 06351, Republic of Korea; neurokong@gmail.com; 5 Department of Neurosurgery, Sungkyunkwan University School of Medicine, Seoul 06351, Republic of Korea

**Keywords:** hemifacial spasm, neurovascular compression, vein, cisternal

## Abstract

The purpose of this study was to investigate the outcome of microvascular decompression (MVD) in patients with hemifacial spasm (HFS) who have no definite radiological neurovascular compression (NVC). Sixteen HFS patients without radiological NVC on preoperative MRI underwent MVD surgery. The symptoms were left-sided in fourteen (87.5%) and right-sided in two patients (12.5%). Intraoperatively, the most common vessel compressing the facial nerve was the AICA (8, 44.4%), followed by arterioles (5, 27.8%), veins (4, 22.2%), and the PICA (1, 5.6%). The most common compression site was the cisternal portion (13, 76.5%) of the facial nerve, followed by the REZ (4, 23.5%). One patient (6.3%) was found to have multiple NVC sites. Arachnoid type (7, 50%) was the most common compressive pattern, followed by perforator type (4, 28.6%), sandwich type (2, 14.3%), and loop type (1, 7.1%). A pure venous compression was seen in two patients, while a combined venous-arterial “sandwich” compression was detected in two patients. Symptom improvement was observed in all of the patients. Only one patient experienced recurrence after improvement. Based on our experience, MVD surgery can be effective for primary HFS patients with no definite radiological NVC. MVD can be considered if the patient shows typical HFS features, although NVC is not evident on MRI.

## 1. Introduction

Hemifacial spasm (HFS) is a chronic movement disorder characterized by irregular, involuntary, and recurring contractions of facial muscles innervated by the ipsilateral facial nerve [1]. It usually initiates from the orbicularis oculi muscle to the orbicularis oris muscle, buccinator muscle, and/or platysma [2]. HFS can result in considerable impairment as it leads to bothersome and distracting twitches and strong spasms, as well as involuntary eye closure, causing a loss of binocular vision. This compressive condition rarely resolves spontaneously [3,4,5,6,7,8,9,10]. The clinical course of HFS is usually characterized by a progressive accentuation of the symptoms. The most common cause of HFS is compression of the facial nerves by the anterior inferior cerebellar artery (AICA) or the posterior inferior cerebellar artery (PICA), most often at the root exit zone (REZ) in the immediate vicinity of the brainstem, while venous conflicts are very rare [11] Multiple compression sites, involving more than two offending vessels, are present in up to 40% of cases [11]. In addition, only in rare cases is HFS caused by NVC of the cisternal segment of the facial nerve [4,12].

Since Gardner et al. first proposed microvascular decompression (MVD) for HFS in 1962, HFS has been considered a surgically curable condition [13] In 1977, Jannetta et al. established the modern MVD operation and later popularized it as a potentially curative treatment for HFS through a number of publications [14,15,16,17]. It has been shown to completely relieve spasms induced by neurovascular compression (NVC) at the REZ, achieving symptom relief in 86% to 97% of patients with a low incidence of morbidity [4,18,19,20,21,22].

High-spatial-solution magnetic resonance imaging (MRI), especially three-dimensional (3D) T2-weighted images, has become essential for excluding a spasm of secondary etiology caused by a cerebellopontine angle (CPA) tumor, inflammation, and demyelinating condition, as well as for identifying NVC and characterizing it [3,7,23]. It may also be useful to the localization of missed NVC when symptoms are not relieved after MVD surgery [3]. To the best of our knowledge, there have been no published data regarding MVD surgery for HFS without evidence of radiological NVC. The aim of this study was to investigate the outcome of MVD in those without definite NVC on MRI.

## 2. Materials and Methods

A total of 5112 patients underwent MVD for HFS between April 1997 and December 2022 at a tertiary institution. HFS was diagnosed based on its typical clinical features. Those with the presence of definite offending vessels on preoperative MR imaging were excluded. During the study period, a total of 16 patients were identified to have no identifiable NVC on MRI. Each patient’s age of onset, symptom laterality, symptom duration, MR images, operative records, clinical outcome, and postoperative complications were retrospectively reviewed. This retrospective study was reviewed and approved by Konkuk University Medical Center Institutional Review Board.

All HFS patients underwent MRI examination using a 3.0 T MR scanner (Achieva, Philips Medical Systems, Best, The Netherlands) with an 8-channel sensitivity-encoding (SENSE) head coil. According to our MR imaging protocol for HFS, 3D heavily T2-weighted fast imaging sequence, so-called MR cisternography (MRC), where the low signal intensity of the nerves and vessels contrasts with the bright signal intensity of background cerebrospinal fluid (CSF), was always undertaken to evaluate the complex relationship between the nerves and vessels. The sequence was displayed in any planes by multiplanar reformation (MPR) to improve the precision of evaluation. In addition, MPR of 3D T2 volumetric isotropic T2-weighted acquisition (VISTA) and 3D fluid-attenuated inversion recovery VISTA images were obtained (axial and oblique coronal). Three-dimensional time-of-flight (TOF) MR angiography (MRA) was also obtained to visualize the offending arteries, although small arterioles are difficult to see consistently with this technique. All MR images were reviewed by a dedicated neuroradiologist. 

The interpretation of microanatomical observations under microscopic guidance relied solely on the senior author (K.P.) to reduce any discrepancies between observers. The compressive patterns were classified into 6 types: loop type (the vascular loop itself without any contributing factors creating the compression), arachnoid type (thick arachnoid trabeculae between the vessel and the brainstem causing the vessel to become tightly tethered to the nerve), perforator type (perforating arteries from the compressing vessel causing compression by tethering the vessel to the brainstem), branch type (the nerve is caught between the compressing vessel and its branch), sandwich type (the nerve is sandwiched between 2 different vessels independently), and tandem type (one vessel compresses another, which, consequentially, compresses the nerve) [20]. Postoperative facial nerve function was evaluated using House–Brackmann (HB) grading [24]. 

All surgeries were performed by a senior neurosurgeon. Details of the MVD technique for HFS have been described previously [5,25,26,27,28,29]. With the patient in a park-bench position under general anesthesia, a “lazy S” skin incision was made at the retroauricular region, and the suboccipital muscles were divided with a Bovie cautery. A conventional retrosigmoid approach was then performed after a lateral suboccipital craniectomy, which was limited to exposing the medial edge of the sigmoid sinus. The mastoid air cells were securely sealed with a thin layer of bone wax to avoid postoperative CSF leakage if they were opened. After the dura was opened, the CSF was adequately drained from the lateral cerebellomedullary cistern. Approaching the jugular foramen was always performed in the first place. Dissection around lower cranial nerves using a micro-bayonet or micro-scissors was performed up to their most medial and superior parts. Initial observation of NVC site without excessive arachnoid dissection or cerebellar shifting is important to localize the offending vessel. After the flocculus was gently retracted, the REZ and cisternal portion of the entire facial nerve were thoroughly examined in 360 degrees to identify and determine the offending vessels, including abutting small veins. The labyrinthine artery should be carefully preserved during inspection near the internal auditory canal (IAC) to preserve hearing; iatrogenic injury and vasospasm often occur following manipulation. Given that NVC is known to commonly occur ventrocaudal to the REZ, the facial nerve was reached from below through an infrafloccular route [30,31]. An interposition technique was predominantly used by placing Teflon felt (Bard PTFE Felt Pledget; Bard Peripheral Vascular, Tempe Inc., Tempe, AZ, USA) at the suspicious NVC sites according to the size of the affected offending vessel or anatomical working space. A transposition technique was also used as needed, especially in cases of HFS with offending vessels harboring a few perforators. At the end of the decompression, Teflon felt pledgets were immobilized using a few drops of fibrin glue for after decompression of the facial nerve. 

Intraoperative brainstem auditory evoked potential and facial electromyography monitoring were routinely performed from the time of administration of general anesthesia until the time of dural closure. Our technique for intraoperative monitoring has been described in detail previously [32,33,34,35]. Lateral spread response (LSR) was used to confirm the adequacy of facial nerve decompression. A postoperative CT scan was immediately obtained before extubation to rule out the presence of intracranial hemorrhage. The patients were usually discharged 5 days after surgery.

## 3. Results

**Figure 1:**
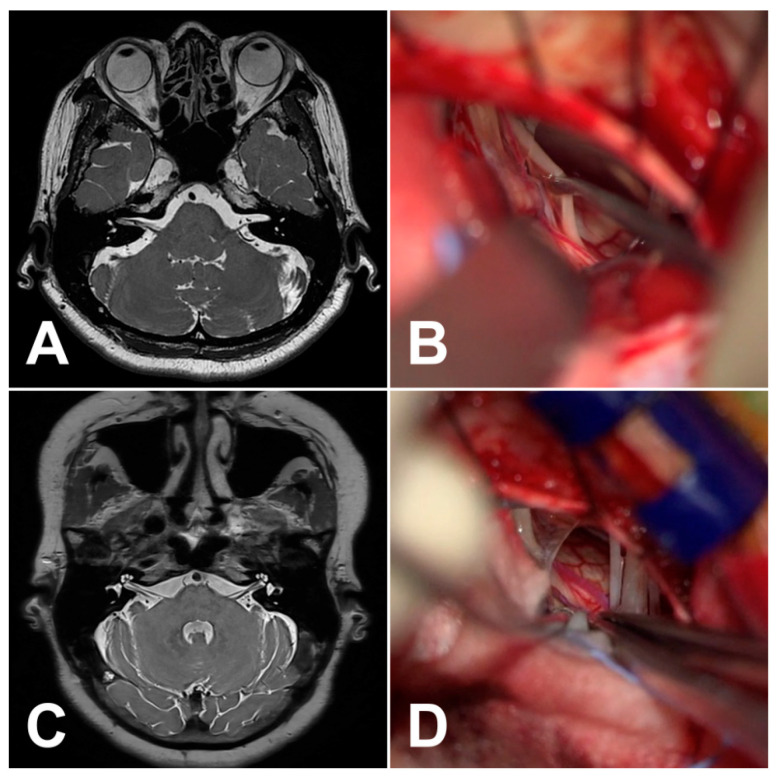
(**A**) Normal 3D MR cisternography (MRC) using 3D VISTA of case 15 with right HFS. (**B**) Intraoperative image showing no NVC at the root exit zone. A combined venous-arterial sandwich compressive type was decompressed at the cisternal segment of the facial nerve. After the small arteriole was initially decompressed, LSR disappeared immediately after the vein was decompressed. (**C**) Normal 3D MRC using 3D VISTA of case 16 with right HFS. (**D**) Intraoperative image showing no NVC at the root exit zone. After the REZ was initially explored 360 degrees, LSR disappeared following the decompression of the small culprit AICA in the form of arachnoid compressive type at the distal, cisternal segment of the facial nerve near the IAC.

The 16 HFS patients without radiological NVC on preoperative MRI underwent MVD surgery (Table 1). Representative cases, including MR images and microscopic views, are shown in Figure 1. The median age at the time of diagnosis was 60 years (range 44–71 years). The median follow-up period was 12 months (range 6–60 months). There were fourteen female (87.5%) and two male (12.5%) patients. The median preoperative duration of symptoms was 37 months (range 4–240). The symptoms were left-sided in fourteen (87.5%) and right-sided in two patients (12.5%). Intraoperatively, the most common vessel compressing the facial nerve was the AICA (8, 44.4%), followed by arterioles (5, 27.8%), veins (4, 22.2%), and the PICA (1, 5.6%). The most common compression site was the cisternal portion (13, 76.5%) of the facial nerve, followed by the REZ (4, 23.5%). One patient (6.3%) was found to have multiple NVC sites. Arachnoid type (7, 50%) was found to be the most common underlying compression mechanism, followed by perforator type (4, 28.6%), sandwich type (2, 14.3%), and loop type (1, 7.1%). A purely venous compression was seen in two patients, while a combined venous-arterial “sandwich” compression was detected in two patients. In 12 patients (75%), LSR disappeared after facial nerve decompression, whereas in two patients (12.5%), it disappeared before the decompression; partial resolution of LSR was noted in one patient (6.2%), and LSR was not detected throughout the operation in one patient (6.2%).

In this series, postoperative improvement of HFS was observed in 15 out of the 16 patients (93.8%); out of these, fourteen patients experienced immediate symptom improvement after surgery, and one patient showed delayed relief of symptoms 11 months after surgery. The remaining patient (6.2%) experienced recurrence after transient improvement following surgery. Four patients (25%) experienced delayed ipsilateral facial palsy, with three having HB grade 3 and one with HB grade 4. All of these patients eventually improved with prednisolone (1 mg/kg/day) and acyclovir (30 mg/kg/day) for at least two weeks. No permanent hearing loss occurred in any cases. In addition, there were no cases of postoperative infection, hemorrhage, or mortality in this series.

## 4. Discussion

Before the advent of modern 3D imaging techniques, the MVD procedure was an exploratory surgery through a retrosigmoid craniotomy, aiming to discover intraoperative evidence of compression on the symptomatic nerve [36,37]. The development of neuroimaging techniques has greatly improved the detection of NVC, especially at the REZ level [38]. The decision as to whether MVD is required has been based on MRI and MRA, both of which can be used to determine if there is arterial compression and exclude the secondary causes of HFS before surgery [39]. Given that MVD is basically functional surgery, however, it is not common to recommend MVD for HFS patients without radiological NVC. Such an absence of radiological NVC in the context of primary HFS has not been well described in the literature. In this study, we investigated the outcomes of MVD for those without definite radiological NVC. Our results showed excellent effectiveness in those patients treated with MVD.

The pathogenetic hypothesis that HFS is attributed to NVC was initially proposed in 1947 [40]. NVC is the most common cause of aberrant excitation, playing a central role in the pathogenesis of HFS [10]. Jannetta et al. first demonstrated that vascular compression to the REZ of the facial nerve was the cause of HFS [17]. Although it has been established that vascular conflict generates aberrant excitation of the facial nerve, the exact physiologic mechanism of excitation has not been fully elucidated [41]. There have been two main leading hypotheses, one “peripheral”, assuming that ephaptic cross-transmission occurs between the affected facial nerve fibers at the location of the vascular compression; and the other, “central”, assuming that primary HFS is caused by hyperactivity at the level of the motor nucleus of the facial nerve [13]. It is most vulnerable at the REZ of the facial nerve where the central glial myelin transitions into the peripheral myelin created by Schwann cells, as a result of the absence of either central glial myelin or peripheral myelin [19]. 

The preoperative identification of NVC via high-spatial-resolution neuroimaging is likely to result in a greater likelihood of symptom resolution following MVD surgery [37]. In this regard, conventional MRI is not sufficient for a reliable interpretation of the complex neurovascular anatomy of the CPA, whereas high-spatial-resolution MRI provides anatomic details of the entire intracranial segment of the facial nerve, including proximal (REZ), middle (between the REZ and the IAC), and distal (around the IAC) [3,42]. As described in the Materials and Methods section, the application of the combination of high-resolution MRI sequences utilizing 3D imaging techniques, including 3D T2-weighted imaging with 3D TOF MRA and 3D contrast-enhanced T1-weighted sequences, allows for the preoperative evaluation of the anatomical course of the offending vessels and other corresponding vessels within the cisterns in the posterior fossa [43,44]. It is also helpful for the preoperative exclusion of a secondary HFS and to searching for and characterizing the NVC [23]. Muller et al. reported that the visualization of the trigeminal nerve using balanced steady-state free precession or fast spin echo sequences, in combination with TOF MRA, enables an optimized delineation of arterial and venous NVCs and may allow for a more reliable differentiation between veins and arteries [44]. Sekula et al. proposed that the sensitivity of thin-slice T2-weighted MRI is high enough that appropriate counseling should be given to clinically favorable surgical candidates without evidence of vascular compression [45]. They pointed out, however, that the specificity of the T2-weighted MRI sequence is low enough that it should not be used to justify operations in clinically unfavorable candidates. Although the aforementioned neuroimaging studies should be interpreted taking into account clinical features, in this series, all patients had no NVCs on preoperative MRI despite having typical HFS features. Fifteen of the sixteen patients (93.8%) had relief of spasm after surgery, which is comparable to the reported overall spasm-free rate ranging from 86% to 96% after MVD in HFS, although the number of treated patients in this series was very limited [4,19,46,47,48]. Our results suggest that MVD surgery can be considered if the patient shows typical HFS features despite the absence of radiological evidence of NVC. In other words, the accurate diagnosis of clinical symptoms of HFS at initial presentation is of utmost importance in making a decision whether to recommend MVD. Also, although primary HFS is mostly associated with a vascular compression of the facial nerve at its REZ, based on our experience, it is recommended that the entire facial nerve be thoroughly inspected 360 degrees from the REZ to the cisternal and distal portions near the porus acusticus and the medial and lateral sides, in order to identify the culprit vessels that are unseen on high-resolution MRI. The operator should explore all relevant vasculature along the facial nerve in the cerebellopontine cistern, including small arterioles and veins, and completely decompress any offending vessels from the facial nerve. We have reported previously that in six (28.6%) and ten (47.6%) out of twenty-one of repeated MVD for HFS, the previously missed culprits were found to compress the cisternal portion and to be medially located to the facial nerve (six at the AICA and two at a vein), respectively [29]. In this study, the cisternal portion of the facial nerve was found to be the most common compression site in HFS with no evidence of radiological NVC.

In patients undergoing MVD, the AICA and the PICA are the most common causative vessels, followed by the vertebral artery, while the internal auditory artery and other small arterioles can also be involved in some instances [3]. On the other hand, veins at the REZ of the facial nerve can also generate symptomatic nerve compression [49]. Venous compression in HFS has been reported in the literature, although it is rare [8,9,19,47,50,51,52]. The veins of the middle cerebellar peduncle and of the pontomedullary sulcus are the common veins in the vicinity of the REZ of the facial nerve [53,54,55]. The offending veins were reported to be involved alongside the artery in 0.7–7.9% or as the only offender in 0.4–5.5% [56]. Toda et al. found that while the cure rates of venous compressions (80%–100%) were comparable to those of arterial compression (87%), long-term recurrences occurred significantly more in cases with both arterial and venous compressions (60%) compared to those with arterial compression alone (3%) and those with venous compression alone (0%; *p* < 0.001) [9]. In some instances, coagulation and division of the compressing vein may be inevitable for the decompression of the facial nerve; thus, the complications secondary to venous sacrifice in the CPA, such as venous congestion and infarction, should be always kept in mind [38,44] In this series, sole venous compression was observed in two patients, while a combined venous-arterial sandwich compressive pattern was seen in two patients. In the latter cases, LSR resolved completely after medially located veins were decompressed. 

The site of vascular compression in HFS is located at the REZ in 95% of the cases, and in 5% distally at the cisternal or the intrameatal portion of the root as the sole conflict or in addition to one at brainstem/REZ [57]. Only a few studies have reported NVC at the distal, cisternal portions of the facial nerve [58,59,60,61,62]. In particular, the cisternal portion of the facial nerve in HFS has been emphasized in the context of failed MVD surgery [62]. The mechanism of HFS caused by the compression of cisternal portions of the facial nerve has been unknown [61]. Additionally, the AICA has been reported to be the main distal offending vessel causing HFS [58,62]. In the current study, however, the cisternal portion of the facial nerve was found to be the most common compression site in 13 of the 16 cases, compared to the REZ.

Lastly, in this study, the arachnoid type was found to be the most common compressive pattern in HFS patients with no evidence of radiological NVC. In this type of compression, unlike the loop type, achieving a mobilization of the causative vessel is challenging unless there is adequate release of the arachnoid tethering between the culprit vessel and the REZ. We have previously demonstrated that the thick arachnoid trabecula was the contributing factor that forced the vessel to compress the REZ [20]. Although it is defined by a compression where the offending vessel adheres tightly to the REZ by thickened arachnoid trabeculae, our results showed that this compression type was also found to be involved in compression at the cisternal portion of the facial nerve. Some studies have stated that the complete separation and dissection of arachnoid membranes can lead to sufficient MVD for HFS. Similatly, El Refaee et al. first reported two cases of HFS induced by strangulation of the facial nerve by arachnoid bands without any NVC at the REZ; in one patient, the facial nerve was anteriorly displaced without definite NVC on MRI, while in both patients, the spasm immediately disappeared after dissecting and cutting the arachnoid bands [8].

The limitations of this study include its retrospective study design, which lacks the rigor of a prospective, randomized controlled trial, and the small number of treated patients with a relatively short follow-up period. Although generalizing the findings of this study is difficult, we believe that they hold clinical significance because the 16 patients were selected from a pool of 5112 patients who underwent MVD surgery for HFS during the study period. Further investigation with a large sample size and long-term follow-up should be warranted to clarify the role of MVD for HFS patients without radiological NVC and to evaluate the long-term outcomes and recurrence rates in these patients. The inclusion of a control group should also be considered in future studies in order to assess the effectiveness of MVD in cases with the absence of radiological NVC. 

## 5. Conclusions

A series of 16 consecutive HFS patients with no evidence of radiological NVC were reviewed in this study. Our results demonstrated excellent effectiveness and safety of MVD for HFS patients without radiological NVC. Based on our experience, MVD surgery can be effective for primary HFS patients without definite radiological NVC. Thus, MVD can be considered if the patient exhibits typical clinical features of HFS, although NVC is not evident on MRI.

## Figures and Tables

**Table 1:** Characteristics and findings in 16 HFS patients without radiological NVC.

Case No.	Age (Years, Sex)	Side	Symptom Duration	Offending Vessel	Compression Site	Compression Type	Outcome	Recurrence	Complication	FU
1	44, F	Left	24	Arteriole	Cisternal (distal)	Arachnoid	Partial	No	No	60
2	69, F	Left	240	AICA	Cisternal	Arachnoid	Complete	No	Transient facial palsy (HB Gr3)	12
3	71, F	Right	36	AICA	Cisternal	Arachnoid	Complete	Yes	No	56
4	48, F	Left	38	Vein	Cisternal	NA	Complete	No	Transient facial palsy (HB Gr3)	40
5	56, F	Left	40	Arteriole	Cisternal	Arachnoid	Complete	No	No	12
6	58, M	Left	32	Arteriole	Cisternal	Arachnoid	Complete	No	No	12
7	58, M	Left	6	Vein	REZ	NA	Complete	No	Transient facial palsy (HB Gr3)	12
8	70, F	Right	12	AICA	REZ (medial)	Arachnoid	Complete	No	No	12
9	55, F	Left	4	AICA	Cisternal (medial)	Perforator	Complete	No	No	12
10	64, F	Left	84	Arteriole	Cisternal (medial)	Loop	Partial	No	No	6
11	53, F	Left	36	AICA	Cisternal	Perforator	Complete	No	No	12
12	62, F	Left	60	AICA	REZ (medial)	Perforator	Complete	No	No	24
13	68, F	Left	24	AICA	Cisternal (between CN VII and CN VIII)	Perforator	Complete	No	Transient facial palsy (HB Gr4)	24
14	63, F	Left	84	PICA (small branch) + vein	REZ + cisternal	Sandwich	Complete	No	No	24
15	62, F	Right	240	Arteriole + vein	Cisternal	Sandwich	Complete	No	No	12
16	57, F	Right	72	AICA	Cisternal (distal)	Arachnoid	Complete	No	No	12

## Data Availability

The data presented in this study are available on request from the corresponding author.
